# Cultivar Grain Yield in Durum Wheat-Grain Legume Intercrops Could Be Estimated From Sole Crop Yields and Interspecific Interaction Index

**DOI:** 10.3389/fpls.2021.733705

**Published:** 2021-10-14

**Authors:** Bochra Kammoun, Etienne-Pascal Journet, Eric Justes, Laurent Bedoussac

**Affiliations:** ^1^AGIR, Univ Toulouse, INRAE, Castanet-Tolosan, France; ^2^ARVALIS—Institut du Végétal, Paris, France; ^3^LIPME, Univ Toulouse, CNRS, Castanet-Tolosan, France; ^4^AGIR, Univ Toulouse, ENSFEA, INRAE, Castanet-Tolosan, France

**Keywords:** cereal, pea (*Pisum sativum* L.), durum wheat (*Triticum turgidum* L.), Faba bean (*Vicia faba* L.), complementarity, competition, model

## Abstract

Ensuring food security for a world population projected to reach over nine billion by 2050 while mitigating the environmental impacts and climate change represent the major agricultural challenges. Diversification of the cropping systems using notably cereal–legume mixtures is one key pathway for such agroecological intensification. Indeed, intercropping is recognised as a practice having the potential to increase and stabilise the yields in comparison with sole crops while limiting the use of inputs notably when species exploit resources in a complementary way. However, predicting intercropped species grain yield remains a challenge because the species respond to competition through complex genotype x cropping mode interactions. Here, we hypothesised that the grain yield achieved by a cultivar in low nitrogen input durum wheat–grain legume intercrops (ICs) could be estimated using a few simple variables. The present work is based on a 2-year field experiment carried out in southwestern France using two durum wheat (*Triticum turgidum* L.), four winter pea (*Pisum sativum* L.), and four winter faba bean (*Vicia faba* L.) genotypes with contrasting characteristics, notably in terms of height and precocity, to explore a wide range of durum wheat–grain legume phenotypes combinations to generate variability in terms of yield and species proportion. The major result is that the yield of durum wheat–grain legume IC component in low nitrogen input conditions could be correctly estimated from only three variables: (i) wheat cultivar full density sole crop (SC) yield, (ii) legume cultivar half density sole crop (SC½) yield, and (iii) an indicator of legume cultivar response to interspecific competition. The latter variable, the interspecific interaction index (IE), reveals cultivars' competitive abilities and tolerance to competition. However, to propose generic IC design and management procedures, further mechanistic understanding is required to better understand the links between tolerance to interspecific competition and cultivar phenotype characteristics. In particular, a special emphasis on the grain legume is needed as their response to interspecific competition appears less predictable than that of durum wheat. Cultivar choice is a key element to optimise the functional complementarity and subsequent IC advantages. This work proposes a simple tool to assist the design of specific breeding programs for cultivars ideotypes adapted to intercropping.

## Introduction

Global agriculture production will have to provide enough food to a world population projected to reach over 9 billion by the year 2050 (FAO, 2010). This challenge is becoming more complex by taking into account the sustainability issues, such as ensuring the availability of resources for the next generations in the context of climate change. These increasing concerns about the environmental impacts and reduction of inputs require a transformation of current cropping systems towards improved efficiency and sustainability (Jackson and Piper, 1989; Vandermeer et al., 1998).

Improving plant diversity within agricultural systems is increasingly recognised as an important pillar of sustainable development (Davies et al., 2009; IAASTD, 2009). Including a larger proportion of legumes has been proposed as a global solution for long by many authors (e.g., Vandermeer et al., 1998; Altieri, 1999). Indeed, exploiting the leguminous symbiotic fixation of atmospheric N_2_ means less nitrogen fertiliser input required (Fustec et al., 2010) contributing to reduced CO_2_ emissions (Nieder and Benbi, 2008) and carbon footprints of agricultural products (Gan et al., 2011). Despite this advantage, grain legumes are less favoured now, because of their supposed low yields and instability related to several factors, such as intolerance to water stress, harvest difficulties due to lodging, diseases, sensitivity to insects, or low competition against weeds.

Intercropping is defined as the growth of two or more species in the same space at the same time (Andrew and Kassam, 1976). Among the species mixtures, the cereal–legume intercrops (ICs) appear as one of the promising levers to enhance the efficiency of the agricultural system in a context of low mineral nitrogen level (Jensen, 1996; Bedoussac and Justes, 2010a; Naudin et al., 2010) and low pesticide inputs, and most notably in organic farming to produce legumes (Malézieux et al., 2009; Lithourgidis et al., 2011; Bedoussac et al., 2015). Compared with the sole crops, intercropping is known to (i) boost crop productivity (Qin et al., 2013), (ii) improve yield stability (Raseduzzaman and Jensen, 2017), (iii) increase cereal grain protein concentration (Lithourgidis et al., 2006; Bedoussac and Justes, 2010b), (iv) favour weeds, pests, and diseases control (Altieri and Liebman, 1986), (v) provide better lodging resistance (Trenbath, 1976), (vi) improve soil conservation (Swift et al., 2004), (vii) improve the use of soil nitrogen (Jensen et al., 2020), or (viii) emit significantly less amounts of greenhouse gases (e.g., Oelhermann et al., 2009; Naudin et al., 2014).

However, optimising the intercropping advantages needs a better understanding of the interactions between: (i) species and cultivars (Ofori and Stern, 1987; Davis and Woolley, 1993; Fukai and Trenbath, 1993; Annicchiarico et al., 2019), (ii) seeding date and density (Davis et al., 1987; Andersen et al., 2007; Barker and Dennett, 2013), (iii) nitrogen availability (Hauggaard-Nielsen, 2001; Corre-Hellou et al., 2006; Bedoussac and Justes, 2010a; Tosti and Guiducci, 2010) altogether in interaction with (iv) climatic and biotic conditions.

Regarding the choice of cultivars within each species, the competitive ability of an IC component is related to some genetic and phenotypic characteristics of cultivars, such as the height and growth dynamics (Davis and Garcia, 1983; Elmore and Jackobs, 1984; Cenpukdee and Fukai, 1992; Annicchiarico et al., 2019). However, the cultivars high yielding in the sole crop are not necessarily high yielding when intercropped due to significant interactions between the genotype and cropping mode (Francis et al., 1978; Francis, 1981; Smith and Zobel, 1991) even though Galwey et al. (1986) could show strong correlations between sorghum cultivar characters in sole crop and when intercropped with cowpea. In addition, some authors (Davis and Garcia, 1983; Elmore and Jackobs, 1984; Cenpukdee and Fukai, 1992) concluded that the intercropped cultivars should reach high yielding levels without affecting the growth of the associated species.

Therefore, specific breeding programs for intercropping are needed (Nelson and Robichaux, 1997; Hauggaard-Nielsen and Jensen, 2001; Barillot et al., 2012; Zajac et al., 2013; Annicchiarico et al., 2019). Recent theoretical developments on the relevant breeding schemes for mixed cropping have been proposed (Annicchiarico et al., 2019; Sampoux et al., 2020; Haug et al., 2021). In particular, Annicchiarico et al. (2019) concluded their review indicating that there is a need for well-focused research on the species, individual traits, and topics that have been overlooked by research. However, the identification of suited characters for intercropped cultivars seems a great challenge since multi-specific stands growth results from an unstable dynamic equilibrium depending on the mutual interaction between the species (Francis, 1981; Davis and Woolley, 1993).

Few crop models have been developed to simulate the species mixtures, such as APSIM (Keating et al., 2003) or STICS (Brisson et al., 2003). Unfortunately, according to Gaudio et al. (2019), their use remains limited notably because they are not fully taking into account the interspecific interactions and in particular the trait plasticity that could explain the behaviour of plants in intercropping. It is also clear that we need to improve our understanding of the ecological processes and dynamical plant–plant interactions involved in the species mixtures and to identify the most relevant parameters including those related to trait plasticity (Gaudio et al., 2019). Therefore, a new toolbox is required, based on the functional ecological principles and modelling approaches before the behaviour of intercropped couples of cultivars could be predicted from their phenotype characteristics.

Our work is based on a 2-year field experiment with durum wheat (*Triticum turgidum* L.)–winter pea (*Pisum sativum* L.) and durum wheat–winter faba bean (*Vicia faba* L.) using two wheat, four pea, and four faba bean genotypes with contrasting characteristics, notably in terms of height and precocity. This allows exploring a wide range of durum wheat–grain legume phenotypes combinations to generate variability in terms of yield and species proportion. The main objective of the present work is to propose and assess a simple statistical model as a proof of concept. The model design was done to represent the interspecific interactions as a whole to estimate the grain yield achieved by each component in durum wheat–grain legume intercropping considering that it depends on: (i) the cultivars grain yield in sole cropping, (ii) the cultivars response to sowing density when it is different in IC and sole crop, and (iii) the cultivars response to interspecific competition.

## Materials and Methods

### Site, Climate, and Soil

**Figure 1:**
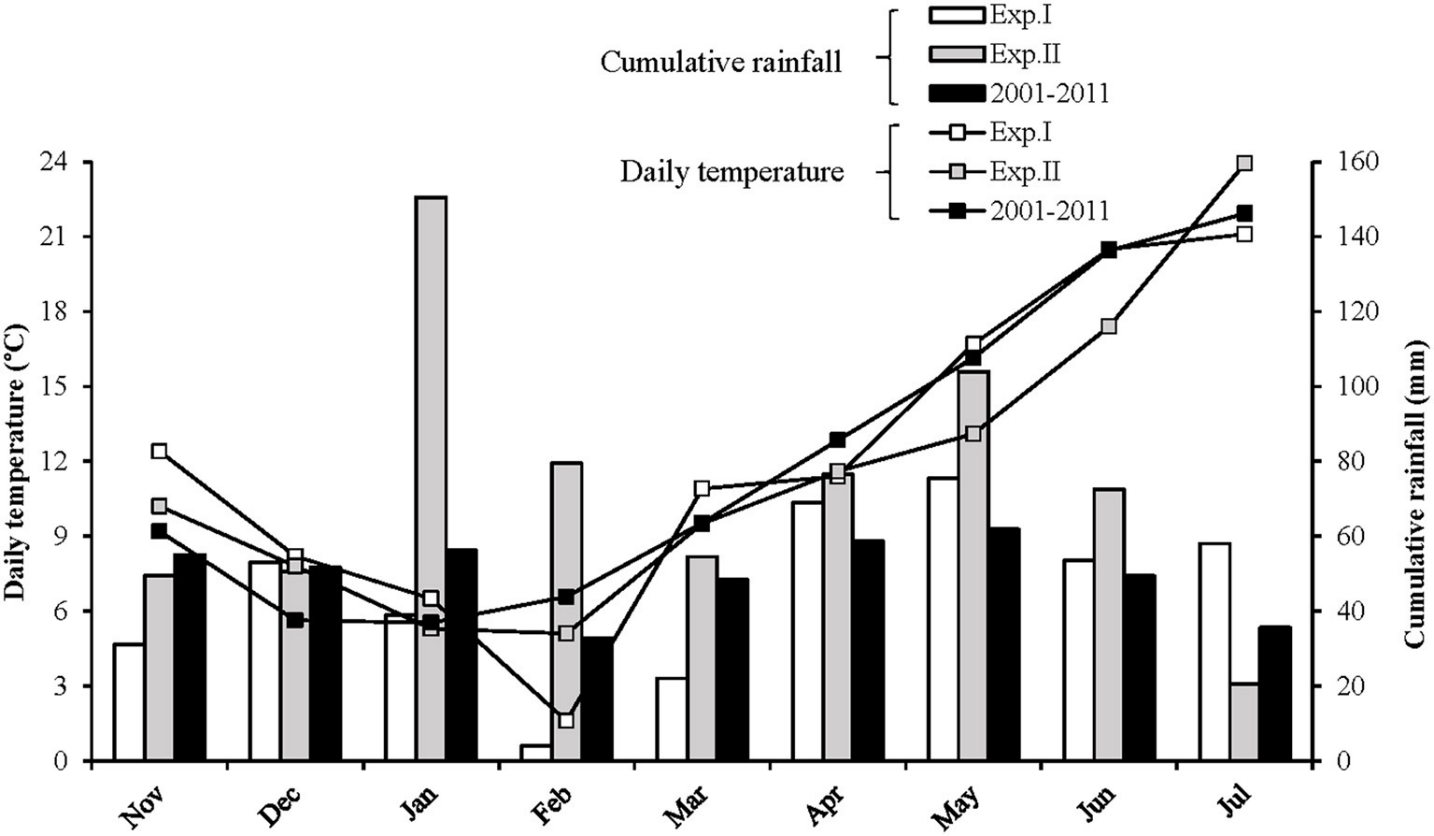
Weather characteristics of the French National Research Institute for Agriculture, Food, and Environment (INRAE) experimental station in Auzeville, southwestern France (43°31′38″N, 1°30′22″E) in 2011–2012 (Exp. I), 2012–2013 (Exp. II), and 2001–2011 (10-year mean).

The experiments were located at the French National Research Institute for Agriculture, Food, and Environment (INRAE) experimental station in Auzeville, southwestern France (43°31′ 38″N, 1°30′22″E) in 2011–2012 (Exp.I) and 2012–2013 (Exp.II). Exp.I was characterised by a very unusual cold period in February (Figure 1) with extremum of −12°C and an average daily temperature of 1.6°C (vs. 6.6°C for the 10-year mean). The rainfall during the growing season (November to July) was (Figure 1) 405 and 658 mm for Exp.I and Exp.II, respectively (vs. 450 mm for the 10-year mean). The rainfall during the February–June period was 224 and 387 mm for Exp.I and Exp.II, respectively (vs. 251 mm for the 10-year mean).

The experiments were carried out on two different experimental fields separated by a dirt road with a clay loamy soil containing 39% clay, 41% silt, and 20% sand in Exp.I and 30% clay, 30% silt, and 41% sand in Exp.II. The field water capacities were 305 and 335 mm on 0–120 cm and soil water content at sowing was 259 and 230 mm for Exp.I and Exp.II, respectively. Total inorganic nitrogen at sowing was 36 and 41 kg nitrogen ha^−1^ on 0–120 cm depth for Exp.I and Exp.II, respectively. For both experiments, the previous crop was sunflower (*Helianthus annuus*).

### Experimental Design

A total of ten cultivars either commercially available or under development were used and chosen within each species for their contrasting height and precocity attributes (Table 1): (i) two of durum wheat (W; L1823 and Sculptur), (ii) four of winter faba bean (F; Castel, Diver, Irena, and Nordica), and (iii) four semi-leafless of winter pea (P) with determinate growth, either insensitive (AOPH10, Isard, and Lucy) or sensitive to photoperiod (Geronimo).

**Table 1:** Characteristics of the cultivars used in this study.

**Species**	**Cultivar**	**Breeder**	**Registration**	**Height**	**Precocity**
Durum Wheat	L1823	INRA	Unregistered	+ +	+ +
	Sculptur	RAGT	2007	+	+ +
Faba Bean	Castel	SCA Epis-Sem	1987	+ +	+ +
	Diver	AgriObtentions	2008	+	+
	Irena	AgriObtentions	2002	+	+ + +
	Nordica	NPZ/Serasem	2010	+ +	+
Winter Pea	AOPH10	AgriObtentions	Unregistered	+ + +	+ +
	Geronimo	Serasem/RAGT	2011	+ +	+
	Isard	INRA/AgriObtentions	2005	+	+ + +
	Lucy	GAE/Serasem	2000	+	+ +

*Class assignment (+: low/late; ++: medium; +++: high/early) for cultivar height at the end of flowering for legumes and the end of heading for wheat and precocity (timing of flowering initiation stage for legumes and of heading initiation stage for wheat)*.

The species and cultivars were grown as (i) full density sole crops (SCs; sown at 336, 29, and 96 grains m^−2^ for wheat, faba bean, and pea, respectively, i.e., 120% of the targeted final plant density), (ii) half density sole crops (SC½; sown at half of the SC density), and (iii) durum wheat–grain legume substitutive ICs (with species mixed on the rows and sown at half the SC density). According to Cruz and Soussana (1997), such a design aims to distinguish and evaluate: (i) interspecific competition when comparing SC½ and IC, and (ii) intraspecific competition when comparing the SC and SC½.

Note that since the faba bean target density was low for SC½ and IC (12 plants m^−2^), it was sown at three times higher density and controlled by manual removal after emergence to obtain a regular plant distribution pattern.

The experimental layout was a randomised split-plot design with three replicates for each combination of cropping treatment, species and cultivar. Each subplot (22.4 m^2^) consisted of 10 rows (14 m-long and spaced 16 cm apart). The fungicide-treated seeds were sown on 14 November 2011 (Exp.I) and 20 November 2012 (Exp.II). No fertiliser was applied while fungal diseases and pests (mainly pea weevils and aphids) were controlled with appropriate pesticides in two applications (one fungicide and one insecticide in Exp.I vs. two fungicides in Exp.II).

The plant densities were measured in each plot on a total of 3 and 10 linear metres (lm) for wheat and legumes, respectively. Aboveground plant parts from the six central rows were mechanically harvested at grain legume maturity for sole cropped legume and at wheat maturity for the IC and wheat sole crop. The samples were dried at 80°C for 48 h, and grain dry weights were determined separating those from IC into wheat and legume.

### Calculations and Statistics

#### Interspecific Interaction Index (IE) for Yield

Interspecific interaction index (IE) allows evaluating the effect of an IC component cultivar on the second IC component by comparing the yield of the second component achieved in IC with that in SC½ (Bedoussac and Justes, 2011) as follows:


$$\begin{array}{l} IE_{Wheat}=\frac{Yield_{ICWheat}}{Yield_{SC1/2Wheat}}\;;\;IE_{Pea}=\frac{Yield_{ICPea}}{Yield_{SC1/2Pea}};IE_{Faba\;bean}\\ \;=\frac{Yield_{ICFaba\;bean}}{Yield_{SC1/2Faba\;bean}} \end{array}$$

where *Yield*_*SC*1/2_ and *Yield*_*IC*_ are the SC½ and IC grain yields per unit area. IE was calculated for each intercrop replicate using the replicate value for the numerator and the mean value over all the three replicates for the denominator to eliminate the variation in the ratio caused by SC½ yield variability.

#### Statistics

The statistical analyses were performed using STATGRAPHICS software (version 15.2.06, Statgraphics Technologies, Inc., VA, USA). All data were tested for normal distribution using the Shapiro–Wilk test and the pairwise comparisons were performed with the least significant difference test (LSD) at a threshold of *p* = 0.05 (Gomez and Gomez, 1984) to compare grain yields among the species, cultivars, and cropping mode. One-tailed *t*-test was applied to compare the means of IE to 1. The prediction interval ellipses were used to describe the area in which a single new observation can be expected to fall with a probability of *p* = 0.90, given that the new observation comes from a bivariate normal distribution with the parameters (means, SDs, and covariance) as estimated from the observed points shown in the plot for ICs (Batschelet, 1981).

## Results and Discussion

### Emergence and Plant Densities

On average for all the treatments and the two experiments, the plant density was close to the objective with observed plant density representing 107% of the expected values for faba bean (105% in IC, 110% in SC, and 104% in SC½), 106% for pea (107% in IC, 106% in SC, and 104% in SC½), and 93% for wheat (96% in IC, 90% in SC, and 94% in SC½).

**Figure 2:**
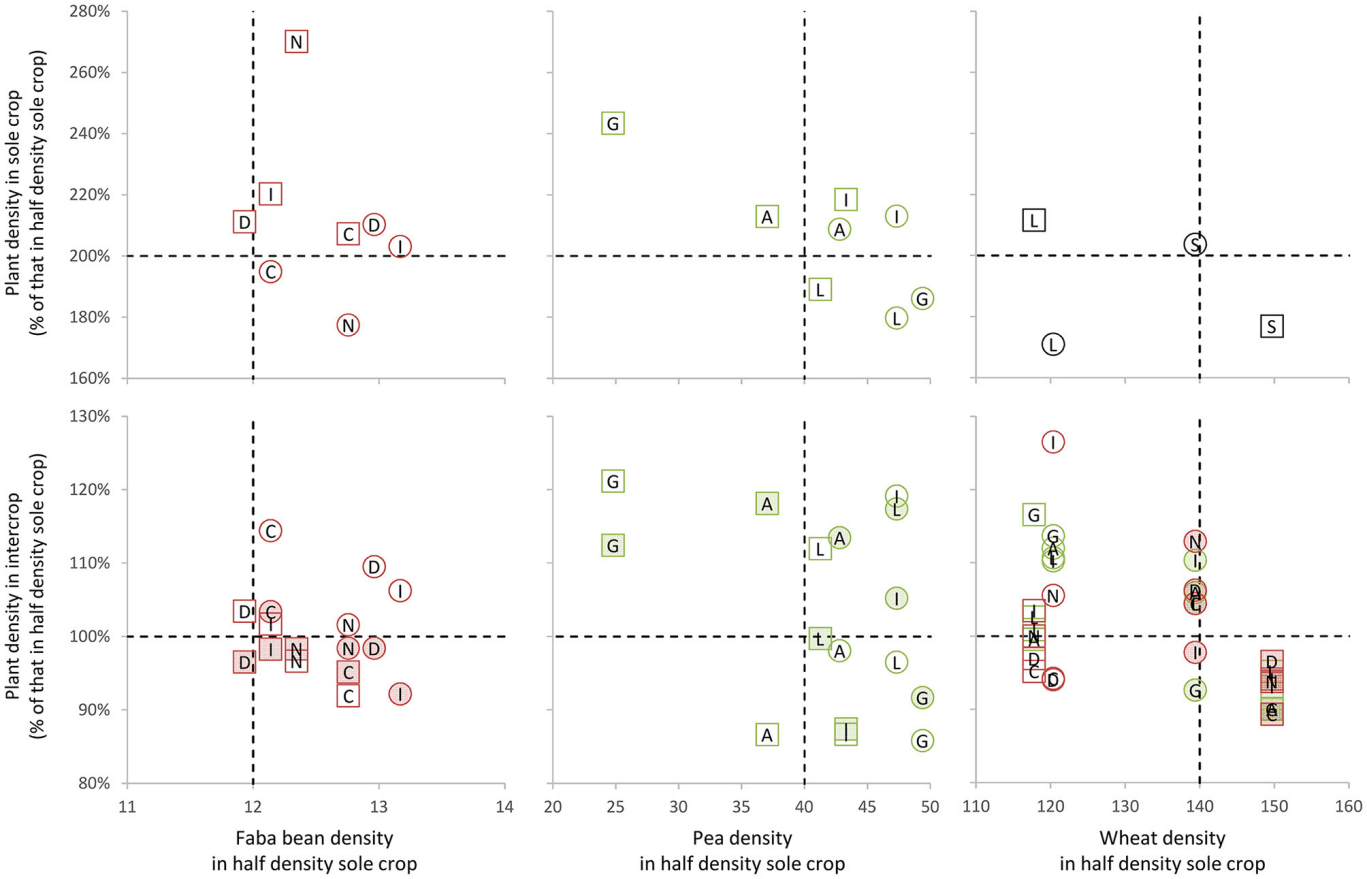
Comparison of the plant densities in intercrop (IC) and sole crop (SC) vs. half density sole crop (SC½). The plant density in IC or SC as a function of that in SC½. The circles correspond to Exp.I and squares to Exp.II. For the ICs, open symbols correspond to L1823 wheat cultivar and closed ones to Sculptur wheat cultivar. Each point corresponds to the means of the three replicates' data. The vertical dotted lines correspond to the targeted density for the SC½. The horizontal dotted lines correspond to the expected densities in IC and sole crop vs. SC½. The letters within the symbols correspond to the first letter of the cultivar for SC vs. SC½ and to the first letter of the associated cultivar in IC vs. SC½.

As illustrated in Figure 2, the plant densities in IC were very similar to those in SC½ (103 ± 13, 100 ± 6, 102 ± 9%, for pea, faba bean, and wheat, respectively). The plant densities in SC were nearly two times higher than those in the SC½ (206 ± 19, 212 ± 25, 191 ± 17%, for pea, faba bean, and wheat, respectively).

No difference was found between the two experiments except for pea with lower plant density in Exp.II than in Exp.I (94 and 117% of the expected density, respectively). A significant difference (*p* < 0.01) was found between the wheat cultivars for both experiments (85 and 101% of the expected density for L1823 and Sculptur, respectively). No difference was found between the faba bean cultivars (104–108% of the expected density) and between the pea cultivars except for Geronimo that had significantly (*p* < 0.001) lower density in Exp.II than the other three cultivars (70 and 102% of the expected density, respectively).

### Species Yields in Durum Wheat–Grain Legume Intercrop Depend Partially on Their Sole Crop Yields

#### Legume Yield in Durum Wheat–Grain Legume Intercrop and Sole Crop

**Figure 3:**
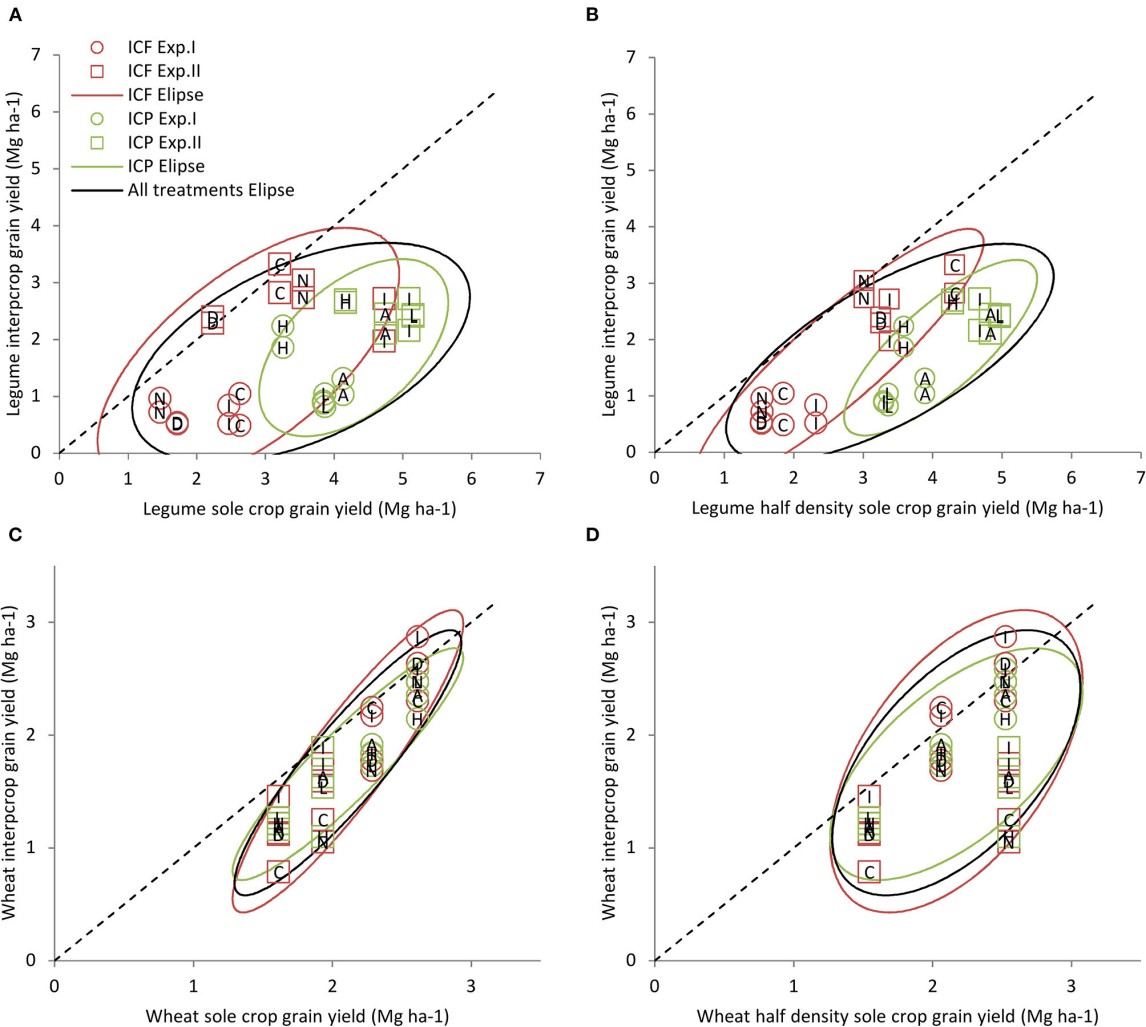
Comparison of yields achieved in IC vs. sole crops for the legumes and wheat. The yield achieved in intercrop by legumes **(A, B)** or wheat **(C, D)** as a function of that in SCs **(A, C)** or SC½ **(B, D)**. Symbols correspond to durum wheat–faba bean intercrop (ICF) (red), durum wheat–pea intercrop (ICP) (green), Exp.I (circles), Exp.II (squares), L1823 wheat cultivar (open), and Sculptur wheat cultivar (closed). The ellipses represent the prediction interval at *p* = 0.90 in red for ICF, in green for ICP, and in black for both the ICF and ICP. Each point corresponds to the mean of three replicates' data.

The legumes grain yield achieved in IC (1.8 Mg ha^−1^) is always significantly lower (*p* < 0.10) than the corresponding SC yield (3.5 Mg ha^−1^), due to both the response to density and interspecific competition, and slightly correlated to it (Figure 3A). Legume grain yield achieved in IC is always significantly lower (*p* < 0.10) than the corresponding SC½ grain yield (3.4 Mg ha^−1^; Figure 3B) due to interspecific competition only.

For both legumes, the SC and SC½ grain yields were similar, underlining the ability of the legumes to compensate for lower densities. In addition, Figures 3A,B show that the behaviours of the two legumes are different as illustrated by distinguishable ellipses. More precisely, pea produced a higher yield than faba bean in both SC (4.3 vs. 2.7 Mg ha^−1^, respectively) and SC½ (4.1 vs.2.7 Mg ha^−1^, respectively) while they yielded similarly in the IC (1.9 and 1.7 Mg ha^−1^, respectively). Therefore, the grain yield loss between SC½ and IC was higher for pea than for faba bean, suggesting that pea is more sensitive than faba bean to wheat competition.

#### Wheat Yield in Durum Wheat–Grain Legume IC and Sole Crop

For the wheat, yield achieved in IC was on average slightly lower than that in SC½ (1.8 vs. 2.2 Mg ha^−1^, respectively) indicating a limited competition by the legume in IC (Figure 3D). Additionally, the wheat yield achieved in IC did not vary on average with the associated species [1.8 and 1.7 Mg ha^−1^ for durum wheat–faba bean intercrop (ICF) and durum wheat–pea intercrop (ICP), respectively] but depended on the associated cultivar (Figures 3C,D).

The wheat yield in SC (2.1 Mg ha^−1^) was similar to that in SC½ underlining the well-known ability of wheat to compensate for low density. The correlation between IC and SC½ wheat grain yield, which reveals only the response to interspecific competition, was worse (Figure 3D) compared with that between IC and SC (Figure 3C) corresponding to the response to both the density and interspecific competition.

These results confirm that the production of a given species in IC cannot be easily predicted neither from its SC or SC½ yields due to the species responses to density and interspecific competition. Therefore, the best varieties for sole cropping are not necessarily the best ones for intercropping, in line with the results obtained by, e.g., Francis et al. (1978) or Smith and Zobel (1991). These results also revealed the limits of the land equivalent ratio (LER; Willey and Osiru, 1972) defined as the relative land area required when growing sole crops to produce, the yield achieved in an IC with the same species proportion. The land equivalent ratio is used in about 11% of the articles on intercropping published between 2000 and 2010 (Bedoussac et al., 2015) and is a relevant indicator to quantify mixture productivity per unit of soil surface for yield as compared with the sole crops. The land equivalent ratio has a didactic virtue to assess the IC performance due to the final balance of competition, complementarity, cooperation, and compensation between the species as named “the 4C approach” by Justes et al. (2021). Nevertheless, the LER cannot identify the intraspecific and interspecific interactions because it is dependent on the sole crop reference and reveals the species responses to both the intraspecific and interspecific competition (Jolliffe, 2000; Bedoussac and Justes, 2011).

### In Durum Wheat–Grain Legume IC, the Yield of a Species Depends Negatively on That of the Associated Species

**Figure 4:**
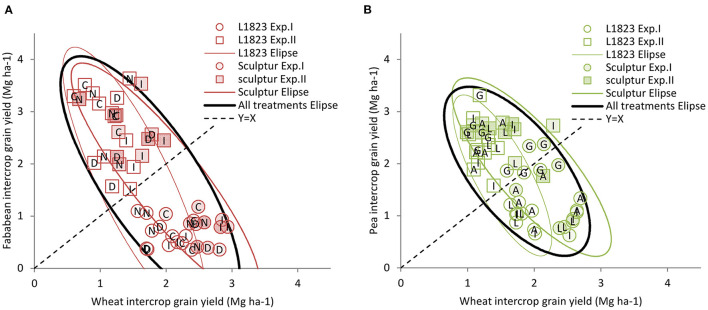
The comparison of legume yield achieved in IC as a function of that of intercropped wheat. Yield achieved in IC by faba bean **(A)** and pea **(B)** as a function of that of the intercropped wheat. The symbols correspond to ICF (red), ICP (green), Exp.I (circles), Exp.II (squares), L1823 wheat cultivar (open), and Sculptur wheat cultivar (closed). The ellipses represent the prediction interval at *p* = 0.90 for ICs with L1823 (thin green or red line), ICs with Sculptur (thick green or red line), and in black for both L1823 and sculptur cultivars. Each point corresponds to a single replicate data.

Figures 4A,B show that the higher the wheat yield in the mixture the lower that of the legume and conversely. Exp.I is characterised by a high wheat yield in the mixture representing 76 and 63% of the total IC grain yield in ICF and ICP, respectively. The converse was observed in Exp.II with the wheat representing only 32 and 36% of the total IC grain yield in ICF and ICP, respectively. Because of the balance between the two associated crops, the total durum wheat–grain legume ICs grain yield remained statistically stable at *p* = 0.05 with ICF and ICP total grain yield varying from ±14 and ±6%, respectively when compared with the average yield over the two experiments.

As the main soil characteristics, such as mineral nitrogen availability, was very similar between the two experiments, the differences in the SC, SC½, and IC yields were very probably explained by the climatic conditions. Indeed, they were drastically different and could explain the inversion of the yield proportions between the two experiments though only partially.

### Climatic Conditions Partially Explain the Inversion of the Yield Proportions

The legumes grain yields (Figures 3A,B) were significantly lower (*p* < 0.01) in Exp.I compared with Exp.II (respectively, 1.0 vs. 2.6 Mg ha^−1^ for IC, 2.9 vs. 4.1 Mg ha^−1^ for SC, and 2.7 vs. 4.1 Mg ha^−1^ for SC½). Conversely, the wheat grain yields (Figures 3C,D) were significantly higher (*p* < 0.01) in Exp.I than in Exp.II (respectively, 2.2 vs. 1.3 Mg ha^−1^ for IC, 2.4 vs. 1.8 Mg ha^−1^ for SC, and 2.3 vs. 2.0 Mg ha^−1^ for SC½). Our results show that the pea yields varied less than those of the faba bean (±19 vs. ±40%, respectively compared with the average yield over the two experiments). This tends to indicate that pea was less sensitive than faba bean to our contrasting climatic conditions.

The Exp.I was indeed characterised by a very cold winter that affected the legume growth more than the wheat growth. In particular, the frost damage symptoms in Exp.I were obvious on both the legumes but especially severe on the faba bean shoots and upper tap roots, resulting in lethality on some plants and several weeks delay before growth resumption. Conversely, the climatic conditions of Exp.II have been favourable to both legumes growth due to the wet spring while it negatively affected the wheat grain yield.

In Exp.I, the wheat proportion in IC is higher than that which would be directly anticipated from the SC yields. This shows that the cereal was more competitive than the legume, in line with a number of reports concluding on the higher competitive ability of the cereal (Jensen, 1996; Hauggaard-Nielsen et al., 2001). However, this dominance potential was probably restricted by the low mineral nitrogen availability which is known as a less profitable situation for the cereal, high nitrogen demanding crop. In Exp.II, the wheat proportion in IC is similar to that which would be directly anticipated from the SC yields. Thus, even if the cereal was potentially more competitive than the legume, the climatic conditions in Exp.II with less favourable conditions to the cereal than to the legume has probably influenced the competitive balance between the crops. These results confirmed that the species production in IC cannot be predicted from the SC yields only, because of complex genotype x cropping mode interactions and species responses to interspecific competition in IC.

### Species Production in Durum Wheat–Grain Legume IC Depends Also on Genotype x Genotype Interactions

Considering durum wheat–grain legume IC yields of the various cultivars of each species, the wheat L1823 had a lower yield compared with Sculptur (Figure 4; 1.5 and 2.0 Mg ha^−1^, respectively on average for ICP and ICF and the two experiments). Surprisingly, the grain legume yield was similar with L1823 and Sculptur (Figure 4; 1.7 and 1.8 Mg ha^−1^, respectively on average over ICP and ICF and the two experiments). Consequently, the whole durum wheat–grain legume IC grain yield was lower (*p* < 0.01) with L1823 than with Sculptur (Figure 4; 3.2 and 3.8 Mg ha^−1^, respectively on average over ICF and ICP and the two experiments).

No significant difference (*p* > 0.10) was observed between the faba bean or pea cultivars for their effect on the total IC grain yield on average over the two experiments (values ranging from 3.2 to 3.6 Mg ha^−1^ for faba bean cultivars and from 3.4 to 3.9 Mg ha^−1^ for pea cultivars; Figure 4). However, the legumes cultivars showed distinct behaviours in their productivity in IC (Figure 4) with: (i) for faba bean cultivars Diver and Irena producing lower yields (1.4 and 1.5 Mg ha^−1^, respectively) than Castel and Nordica (2.0 and 1.9 Mg ha^−1^, respectively) and (ii) for pea cultivars Lucy, Isard, and AOPH10 producing lower yields (1.7 Mg ha^−1^) than Geronimo (2.4 Mg ha^−1^, respectively). Consequently, the wheat grain yield was significantly (*p* < 0.01) higher (Figure 4) when intercropped with Diver and Irena (1.8 and 2.1 Mg ha^−1^, respectively) than with Castel and Nordica (1.6 Mg ha^−1^ for both). Conversely, the wheat grain yield in IC was not affected by the pea cultivars (values ranging from 1.6 to 1.9 Mg ha^−1^; Figure 4).

These results indicate that the total durum wheat–grain legume IC yield and its composition is not only determined by the choice of the two intercropped species but also by each species cultivars, in line with other studies (Davis and Woolley, 1993; Nelson and Robichaux, 1997; Hauggaard-Nielsen and Jensen, 2001). In fact, the cultivars within a species display diverse characteristics which contribute to determining the level of complementarity or competition with the second species in IC, leading to genotype x genotype interactions with the effect of cultivars which can be higher than that of species.

These elements can be illustrated by the high complementarity obtained with the photoperiod-sensitive cultivar Geronimo. This could be the consequence of a delayed and later vegetative growth combined with its more active stem branching (Weller et al., 1997; Lejeune-Hénaut et al., 2008). Indeed, this possibly results in the different times in peak requirements for resources such as nitrogen and light and thus characterising an over-time complementarity situation (Bedoussac and Justes, 2010b). Thus, these results confirm that the species production in IC depends on genotype x genotype interactions which must be taken into account for modelling approaches to estimate the yield achieved in IC from the SC data.

### Interspecific Interaction Index Value Depends on the Associated Species Yield

**Figure 5:**
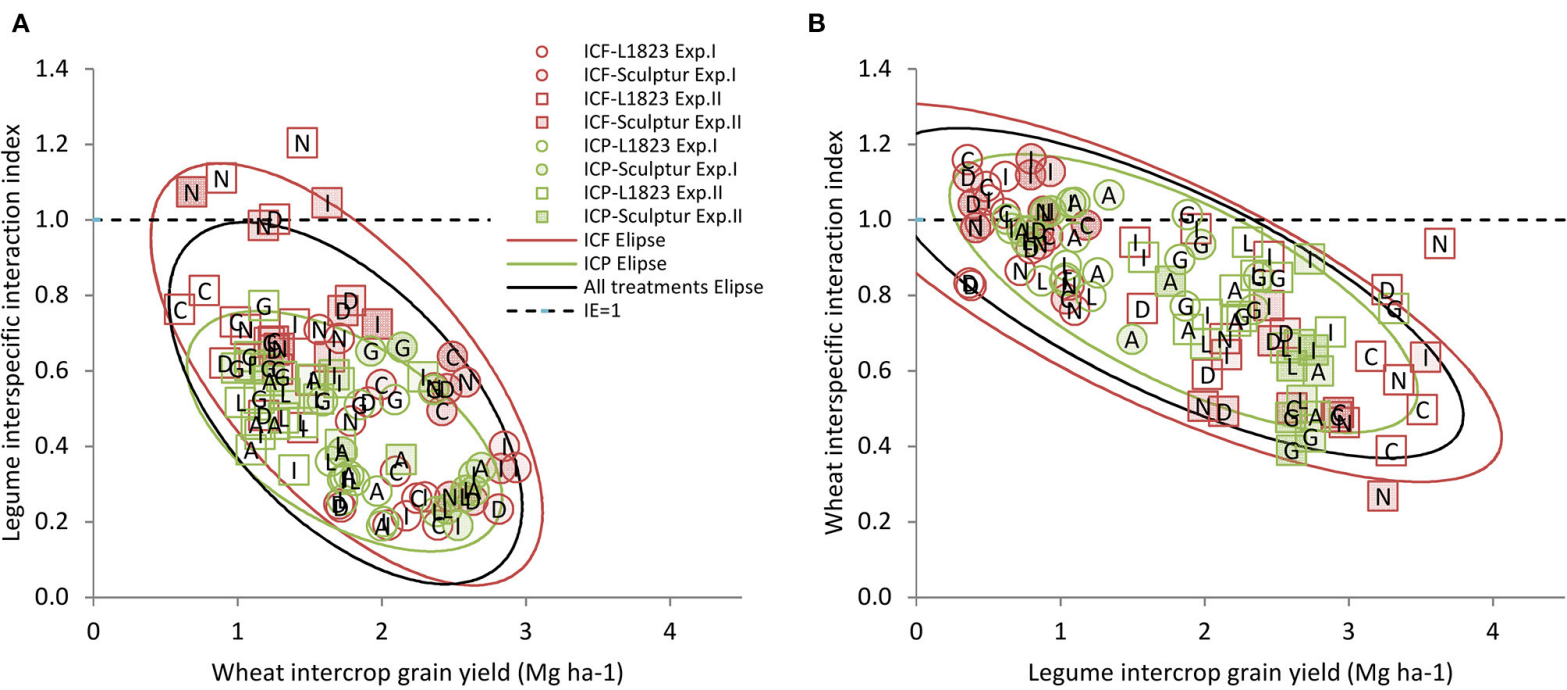
Relationship between interspecific interaction index and the associated species yield. The IE was calculated from the grain yields for legumes **(A)** and for wheat **(B)** as a function of the yield of the associated species (Mg ha^−1^). The symbols correspond to ICF (red), ICP (green), Exp.I (circles), Exp.II (squares), L1823 wheat cultivar (open), and Sculptur wheat cultivar (closed). The ellipses represent the prediction interval at *p* = 0.90 in red for ICF, in green for ICP, and in black for both ICF and ICP. Each point corresponds to a single replicate data. It should be borne in mind the inverse relationship between the IE index values and the levels of interspecific competition effects, respectively, i.e., the lower the IE index value for a species, the stronger its sensitivity to the interspecific competition within the crop stand.

Interspecific interactions can only be relevantly analysed by comparing the ICs with sole crops sown at half-density and not directly with sole crops sown at normal density as for the land equivalent ratio (Bedoussac and Justes, 2011). The IE index is an indicator that compares the production of one component species in IC with its production in SC½. The IE index thus reflects the intensity of the interspecific competition effect on a species. For the three species studied, the IE values were almost always lower than 1 (Figure 5) indicating a lower yield achieved in IC than in SC½ due to interspecific competition. The IE values were also negatively and significantly correlated (*p* < 0.001) with the yield of the associated species (Figure 5). This clearly indicates that the greater the associated species yield, the stronger the interspecific competition effect on the first species, in line, for example, with, Bedoussac and Justes (2010b).

On average for the two experiments, the slopes of ellipses are significantly steeper (*p* = 0.01) for the legumes (Figure 5A) than for the wheat (Figure 5B). This result indicates that the legumes are more affected than the wheat by the increase of the associated species yield. More precisely, for a similar associated species yield, the legume yield loss proportionally to SC½ is higher than for the wheat (IE_Wheat_ > IE_Legumes_) revealing that the legume is more sensitive to the interspecific interactions than the wheat. No difference was found between the slopes of the ellipses for the two legumes (Figure 5A). This signifies that an increase of, e.g., 1 Mg ha^−1^ of wheat yield leads to the same reduction of IE value for both pea and faba bean. However, on average for the two experiments, the IE values of pea were lower than those of faba bean (0.44 vs. 0.59, respectively) for a similar wheat grain yield (1.7 vs. 1.8 Mg ha^−1^, respectively). This indicates that for a similar wheat yield, the pea yield loss in IC compared to SC½ is higher than that of the faba bean (IE_Pea_ < IE_Fababean_), i.e., the pea is more sensitive to the interspecific interactions than the faba bean.

Finally, the response of wheat to interspecific competition was similar irrespective of the legume species as illustrated by the same ellipses slopes values (Figure 5B) and, on average for the two experiments, the same IE_Wheat_ values (0.81 for both ICF and ICP) for a similar legume grain yield (1.9 and 1.7 for both ICF and ICP, respectively).

Considering a simple linear regression between IE and grain yield of the associated species should not mask that the response to the associated species yield increase is certainly not linear. Indeed, for the low yield values, the interspecific competitions are almost null. In such a case and except if facilitation occurs, IC can be considered as an SC½ leading to the IE values close to 1. Such a situation was observed (Figure 5B) in Exp.I with the high IE_Wheat_ values (0.8–1.1) associated with low legume yield (mostly below 1 Mg ha^−1^) while at the same time (Figure 5A) low IE_Legume_ values (0.2–0.6) correspond to the great wheat yield (2–3 Mg ha^−1^) reflecting the strong disequilibrium between the two species. Conversely, Exp.II leads to a situation in which both the wheat and legume had intermediate IE values compared to Exp.I (Figures 5A,B).

In conclusion, an analysis of the relationship between IE and the associated species yield in a variety of situations is an informative approach to determine and compare the competitive abilities and tolerance to the competition of various cultivars within and among durum wheat–grain legume intercropped species. This leads us to formulate the hypothesis that the mean of IE values over all ICs and over the two experiments calculated for a given genotype can be considered as an indicator characterising its global tolerance response to the interspecific competition.

### Estimation of Cultivar Yields in Durum Wheat–Grain Legume ICs From Both the Sole Crop Yields and Average IE Indices

#### Modelling IC Grain Yield

We showed that, under a given set of pedo-climatic conditions, the behaviour of each cultivar in durum wheat–grain legume IC is related to: (i) its growth potential in a pure stand (Figure 3), (ii) its response to the density when that of the pure stand reference is different (Figure 3), and (iii) its response pattern to the interspecific competition (Figures 4, 5) which is related to the growth potential in the pure stand of the associated cultivar (Figure 3).

Therefore, we here formulate the hypothesis that it should be possible to estimate the durum wheat–grain legume IC yield of each intercropped cultivar based on the SC and SC½ yielding of each of the two cultivars and their IE mean values over all durum wheat–grain legume ICs and experiments ($\bar{IE}$) as an indicator of their response pattern to interspecific competition. Note that using the $\bar{IE}$ values avoid a direct and circular mathematical link with the IC grain yield, conversely to the use of IE values calculated as the mean of the three replicates of a given treatment.

An analysis of covariance (ANCOVA) procedure was first applied to test the relationships between the cultivar IC grain yield and the six explanatory variables mentioned above. We added the type of species (legume or wheat) and the legume species (i.e., faba bean or pea) as co-variables to determine if these relations were different among the groups. The ANCOVA showed significant (*p* < 0.01) effects of SC and SC½ grain yields altogether with the $\bar{IE}$ mean values on the grain yield achieved by a cultivar in durum wheat–grain legume IC. Testing species as a co-variable indicated that these relations were different between the wheat and legumes and between the pea and faba bean leading to the following structure of statistical linear “complete” models:

- Durum wheat–pea complete model (ICP_Complete model_):

$$\begin{array}{l} Yield_{P-ICP}=a\times Yield_{SCW}+b\times Yield_{SC1/2W}+c\times \bar{IE_{W}}\;\\ \;+d\times Yield_{SCP}+e\times Yield_{SC1/2P}+f\times \bar{IE_{P}}+g\\ Yield_{W-ICP}=a^{\prime }\times Yield_{SCW}+b^{\prime }\times Yield_{SC1/2W}+c^{\prime }\times \bar{IE_{W}}\\ \;+d^{\prime }\times Yield_{SCP}+e^{\prime }\times Yield_{SC1/2P}+f^{\prime }\times \bar{IE_{P}}+g^{\prime } \end{array}$$

- Durum wheat–faba bean complete model (ICF_Complete model_):

$$\begin{array}{l} Yield_{F-ICF}\;={a\times Yield}_{SCW}+{b\times Yield}_{SC1/2W}+c\times \bar{IE_{W}}\\ \;+{d\times Yield}_{SCF}+{e\times Yield}_{SC1/2F}+f\times \bar{IE_{F}}+g\\ Yield_{W-ICF}=a^{\prime }\times Yield_{SCW}+b^{\prime }\times Yield_{SC1/2W}+c^{\prime }\times \bar{IE_{W}}\\ \;+d^{\prime }\times Yield_{SCF}+e^{\prime }\times Yield_{SC1/2F}+f^{\prime }\times \bar{IE_{F}}+g^{\prime } \end{array}$$

However, such an elevated number of variables precludes practical use. Subsequently, to simplify the model, a multiple regression procedure was applied for the cultivars of wheat, pea, and faba bean separately and considering only three variables (Yield_SCW_, Yield_SC1/2P_ and $\bar{IE_{P}}$for durum wheat–pea; Yield_SCW_, Yield_SC1/2F_, and $\bar{IE_{F}}$for durum wheat–faba bean) resulting in the following linear “simplified” models:

- Durum wheat–pea simplified model (ICP_Simplified Model_):

$$\begin{array}{l} Yield_{P-ICP}\;={a\times Yield}_{SCW}+{b\times Yield}_{SC1/2P}+c\times \bar{IE_{P}}+d\\ Yield_{W-ICP}={a^{\prime }\times Yield}_{SCW}+{b^{\prime }\times Yield}_{SC1/2P}+c^{\prime }\times \bar{IE_{P}}+d^{\prime } \end{array}$$

- Durum wheat–faba bean simplified model (ICF_Simplified Model_):

$$\begin{array}{l} Yield_{F-ICF}\;={a\times Yield}_{SCW}+{b\times Yield}_{SC1/2F}+c\times \bar{IE_{F}}+d\\ Yield_{W-ICF}={a^{\prime }\times Yield}_{SCW}+{b^{\prime }\times Yield}_{SC1/2F}+c^{\prime }\times \bar{IE_{F}}+d^{\prime } \end{array}$$

#### Simplified vs. Complete Model

Considering only three explanatory variables in the simplified model makes the cultivar IC yield fitting more robust and functional with only a slightly lower quality of adjustment than the complete model (RMSE ranging from 0.15 to 0.30 vs. 0.13 to 0.23; Table 2). This confirms that the model fitting quality does not always depend upon its complexity or number of variables. In both the complete and simplified models, wheat IC yield is positively related to that in SC while the IC legume yield is positively correlated to the SC½ yield (Table 2). This is consistent with results described in Figure 3 revealing contrasted responses between the species to plant density. More precisely, it underlines that the legumes are less prone to compensate for a low density than the wheat, which is able to produce more tillers upon favourable pedo-climatic conditions thus leading to quite similar yields in SC and SC½. Because of the balance between the two species in IC, the wheat IC yield is negatively related to the legume SC½ yield and the IC legume yield is negatively correlated to the wheat SC yield.

**Table 2:** Parameters values and adjustment quality for the complete and simplified models.

	**Complete model**				**Simplified model**			
	**ICF Model**		**ICP Model**		**ICF Model**		**ICP Model**	
	**Wheat**	**Faba bean**	**Wheat**	**Pea**	**Wheat**	**Faba bean**	**Wheat**	**Pea**
Yield_SCW_	1.50	−1.54	1.26	−1.73	1.26	−0.50	1.28	0.02
	*p* = 0.02	*p* = 0.04	*p* < 0.0001	*p* < 0.001	*p* < 0.01	*p* = 0.25	*p* < 0.0001	*p* = 0.95
${\text{Yield}}_{\text{SC}\frac{1}{2}W}$	−0.55	−0.31	−0.41	0.19	–	–	–	–
	*p* = 0.26	*p* = 0.59	*p* = 0.25	*p* = 0.58				
IE_Wheat_	−5.71	−12.84	−5.30	−9.13	–	–	–	–
	*p* = 0.33	*p* = 0.09	*p* = 0.18	*p* = 0.03				
Yield_SCLeg_	0.13	−0.01	0.05	−0.66	–	–	–	–
	*p* = 0.18	*p* = 0.93	*p* = 0.88	*p* = 0.07				
${\text{Yield}}_{\text{SC}\frac{1}{2}Legume}$	−0.18	0.51	−0.09	0.29	−0.10	0.89	0.10	0.98
	*p* = 0.33	*p* = 0.04	*p* = 0.74	*p* = 0.32	*p* = 0.44	*p* < 0.001	*p* = 0.45	*p* < 0.001
IE_Legume_	−1.37	2.32	−1.00	1.12	−1.39	3.16	−0.99	4.33
	*p* = 0.11	*p* = 0.04	*p* = 0.39	*p* = 0.32	*p* = 0.08	*p* < 0.01	*p* = 0.08	*p* < 0.001
Constant	5.17	12.93	4.72	13.35	0.19	−1.48	−0.94	−4.15
	*p* = 0.39	*p* = 0.10	*p* = 0.20	*p* < 0.0001	*p* = 0.87	*p* = 0.37	*p* = 0.38	*p* = 0.02
RMSE	0.19	0.23	0.14	0.13	0.22	0.30	0.15	0.22
BIAS	7.1·10^−16^	−5.3·10^−16^	1.6·10^−15^	−1.3·10^−15^	−2.8·10^−17^	−4.6·10^−16^	1.4·10^−16^	−9.5·10^−16^
EF	0.89	0.95	0.91	0.96	0.84	0.90	0.88	0.89

*Yield units are Mg ha^−1^. The level of statistical significance of the associated variable in the models corresponds to that revealed by the multiple regressions*.

**Figure 6:**
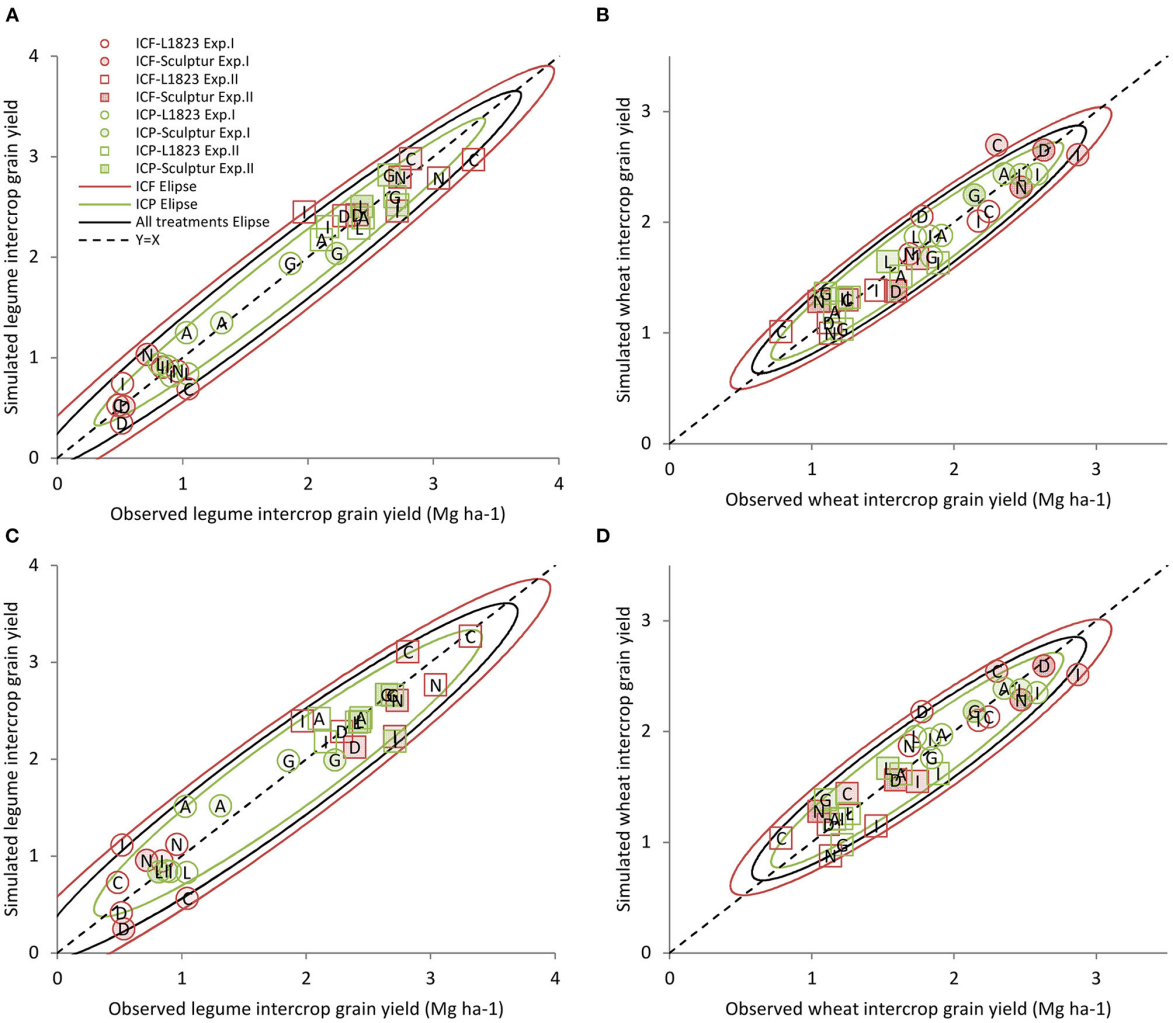
The simulated yield achieved in IC as a function of that observed. Grain yield in IC estimated as a function of that observed considering the complete models with six variables **(A, B)** or the simplified models with three variables **(C, D)** for the two legumes **(A, C)** or the wheat **(B, D)** both distinguishing the pea and the faba bean. Symbols correspond to ICF (red), ICP (green), Exp.I (circles), Exp.II (squares), L1823 wheat cultivar (open), and Sculptur wheat cultivar (closed). The ellipses represent the prediction interval at *p* = 0.90 in red for ICF, in green for ICP, and in black for both ICF and ICP. Each point corresponds to the mean of three replicates' data.

The $\bar{IE_{P}}$ and $\bar{IE_{F}}$variables can be considered as an indicator of the tolerance to interspecific competition in IC of the pea and the faba bean, respectively. They are positively correlated to the IC legume yield because the higher the $\bar{IE_{P}}$ and $\bar{IE_{F}}$values the lower the loss between SC½ and IC legume yields. Oppositely, $\bar{IE_{P}}$ and $\bar{IE_{F}}$are negatively correlated with the IC wheat yield as shown in Figure 6. Indeed, the high $\bar{IE_{P}}$ and $\bar{IE_{F}}$values correspond to low IC wheat yield which mostly indicates a strong competitive effect of the legume, the converse being true for the low $\bar{IE_{P}}$ and $\bar{IE_{F}}$values. This statement is reinforced by the fact that, in the equation for wheat IC yield, the $\bar{IE_{F}}$ parameter was lower than that of $\bar{IE_{P}}$ (−1.39 vs. −0.99, respectively) altogether with the higher $\bar{IE_{F}}$value than $\bar{IE_{P}}$value (0.59 vs. 0.44, respectively). This reflects that the effect of faba bean cultivars on IC wheat grain yield was more significant than that of the pea cultivars.

In our situation, the quality of the simplified model is satisfactory to identify the most important variables explaining both the wheat and legume IC yields for the two experiments with contrasting climatic conditions. However, the potential yield as expressed by the SC and the SC½ yields, for wheat and legumes, respectively, are greatly dependent on the pedo-climatic conditions. Moreover, the $\bar{IE_{P}}$ and $\bar{IE_{F}}$values are strongly dependent on the experiments and particularly on the diversity of genotype x genotype combinations used, making the predictive quality of the model still questionable. For these reasons, there is now a need to understand in a dynamic fashion the link between the $\bar{IE_{P}}$ and $\bar{IE_{F}}$values and the plant characteristics to be able to define relevant phenotypic indicators of the competitive ability of a cultivar.

## Conclusions

Durum wheat–grain legume IC yield and its composition are greatly influenced by species and cultivar choice, and we observed a significant wheat cultivar x grain legume cultivar interaction. This work makes the proof of concept that a simple statistical model could allow predicting the yield of each durum wheat–grain legume IC component from only three simple and easy to measure and calculate variables: (i) the sole crop yields of wheat cultivars, (ii) SC½ yields of legume cultivars, and (iii) an indicator of legume cultivar tolerance to interspecific competition. However, the predictive quality of the model is probably limited and further studies on more diverse genotypes and growing conditions should be conducted to enlarge this finding. The applicability of the model could thus be extended to a variety of typical species × climate × management combinations. Moreover, further mechanistic understanding is required to better evaluate the links between the tolerance to interspecific interactions and the plant phenotype characteristics (traits). Such links will be useful for specific breeding programs of cultivars for intercropping as already pointed out by several authors (e.g., Nelson and Robichaux, 1997; Hauggaard-Nielsen and Jensen, 2001; Annicchiarico et al., 2019) to reveal the plant characters, such as height, leaf area, or root architecture to optimise complementarity between the species.

## Data Availability Statement

The raw data supporting the conclusions of this article will be made available by the authors, without undue reservation.

## Author Contributions

EJ: funding acquisition. BK: data collection and formatting. BK and LB: data analysis. BK, E-PJ, and LB: Writing original draft. BK, E-PJ, EJ, and LB: writing, review and editing. All authors contributed to the article and approved the submitted version.

## Funding

This work was supported in part by the French ANR research programs PerfCom (ANR-09-STRA-11) and MicMac-Design (ANR-09-STRA-06) and by the European Union through the H2020 ReMIX project (Redesigning European cropping systems based on the species mixtures; Grant agreement ID: 727217). BK is a recipient of a PhD grant from the Tunisian government.

## Conflict of Interest

The authors declare that the research was conducted in the absence of any commercial or financial relationships that could be construed as a potential conflict of interest.

## Publisher's Note

All claims expressed in this article are solely those of the authors and do not necessarily represent those of their affiliated organizations, or those of the publisher, the editors and the reviewers. Any product that may be evaluated in this article, or claim that may be made by its manufacturer, is not guaranteed or endorsed by the publisher.

## Abbreviations

SC: full density sole crop
SC½: half density sole crop
IC: intercrop
ICF: durum wheat–faba bean intercrop
ICP: durum wheat–pea intercrop
SCW: full density durum wheat sole crop
SC½W: half density durum wheat sole crop
SCF: full density faba bean sole crop
SC½F: half density faba bean sole crop
SCP: full density pea sole crop
SC½P: half density pea sole crop
IE: interspecific interaction index.
